# Comparison of Titratable Oral Appliance and Mandibular Advancement Splint in the Treatment of Patients with Obstructive Sleep Apnea

**DOI:** 10.5402/2011/581692

**Published:** 2011-06-09

**Authors:** Emel Sari, Steven Menillo

**Affiliations:** ^1^Kasimpasa Military Hospital, Istanbul, Turkey; ^2^Department of Dentistry, Hackensack University Medical Center, Hackensack, NJ 07601, USA

## Abstract

*Objective.* To compare the effect of two intraoral devices (titratable oral appliance-Klearway (KW) and mandibular advancement splint (MAS)) in mild and moderate obstructive sleep apnea (OSA) patients. *Method and Materials.* The study group was comprised of twenty-four adult volunteer patients with OSA. Twelve subjects were fitted with a titratable oral appliance (group KW) protruding the mandible (85% of maximum protrusion). The other 12 subjects received MAS with 75% protrusion of the mandible (group MAS). Baseline, (“0.PSG”), first week (K1.PSG for KW group and M1.PSG for MAS group), and after the first month (K2.PSG for KW group and M2′. PSG for MAS group). *Results.* Both groups produced similar reduction in apnea-hypopnea index (AHI) from baseline till the end of the first week and first month (*P* < .05). However, the success rate of both groups at the end of the first month was found to be statistically different from the success rate of the first week (*P* < .05). The reduction in mean AHI of group KW-moderate (KW-mo) was significantly different from the mean AHI of group MAS-moderate (MAS-mo) at the end of the first month (*P* < .05). *Conclusion.* This study suggests that Klearway appliance was more effective in treating moderate OSA patients than MAS appliance. It was concluded that an appliance that provides 85% mandibular advancement to open the upper airway was more effective in reducing the number of high apneic events during sleep in comparison to the one which provides 75%.

## 1. Introduction

Snoring and obstructive sleep apnea syndrome (OSAS) are common disorders related with the narrowing of the upper airway. Many treatment methods have been tried over the years to treat snoring and obstructive sleep apnea (OSA) [1]. Today, three approaches, namely, nasal continuous positive airway pressure (nCPAP), surgical techniques, and use of intraoral appliances (OAs) seem to be the most effective ones [2–5].

Intra-OAs are indicated in patients with primary snoring or having mild OSA who do not respond to or are not appropriate candidates for treatment with behavioral measures such as weight loss or change of sleep position. On the other hand, OAs have been advised for patients with moderate to severe OSA who cannot tolerate or refuse treatment with nCPAP or subjects who are not suitable surgical candidates [6, 7]. Intra-OAs are worn in the mouth during sleep to prevent the oropharyngeal tissues and the base of the tongue from collapsing and obstructing the airway [8–11].

Mandibular advancement splint (MAS) is a nonadjustable, one-piece appliance (monoblock) and functions to repose and maintain the mandible in a protruded position and vertical opening between 5 and 7 mm during sleep [2, 8, 11–13]. Titratable OA is an adjustable, two-piece appliance. Among these devices, Klearway appliance is the most thoroughly researched OA for treatment of snoring and OSAS [12–16]. Both appliances are mandibular repositioners (MRs) that advance the mandible and tongue base, increasing the space between the base of the tongue and the posterior pharyngeal wall.

 Lawton et al. [15] compared the Twin Block and Herbst mandibular advancement splint in the treatment of patients with obstructive sleep apnea. They found that both appliances were effective on the AHI, snoring frequency, arterial blood oxygen saturation, quality of life, and side-effects. On the other hand, side effects with both appliances were minor and improved in longer term. Kuna et al. [16] compared the polysomnographic results of EMA-T (elastic mandibular advancement) appliance and Klearway in the long-term treatment. They observed that the mean AHI decreased from 17.8 events/h at the baseline to 4.2 events/hr at 6 to 12 weeks of treatment, to 8.2 events/hr after 6 to 12 months of treatment, and to 8.3 events/hr 18 to 24 months later.

Our aim was to evaluate the effects of MAS and Klearway appliance on OSA patients by comparing the AHI results at different time intervals and to investigate patients' short-term compliances.

## 2. Materials and Methods

A total of 24 OSA patients participated in the study. The mean age was 39 ± 4.2. The mean body mass index was 32.3 ± 5.1 kg/m^2^, and the mean neck size was 42 ± 3.5 cm. The patients were referred from the Council of the Sleep-Respiration Disorders of Ege University Medical Faculty Research Hospital with a history and diagnosis of intrusive snoring and obstruction in their sleep, and their history was taken accordingly with the following areas of interest, by the Department of Chest Diseases, Faculty of Medicine, Ege University.

### 2.1. Diagnosis

The results of the patient's history showed that the patients

- had severe snoring problems on the back position and daytime drowsiness;
- witnessed apneas, blockage feeling, perspiration during sleep, and insomnia;
- had morning headaches and dryness in mouth;
- did not have addiction to sedatives, alcohol, or smoking;
- were treated for hypertension and diabetes mellitus Type 2.

Dental examination of the patients consisted of the following: study casts and panoramic radiograph. The temporomandibular joint (TMJ) evaluation such as palpation, auscultation, and evaluation of dentition and occlusion were applied. Muscle palpation and motion range of the jaw, such as maximum opening (40–60 mm) and lateral and protrusive movement (>8 mm) were also evaluated. All of the patients had more than 10 teeth in each jaw and had no symptoms of temporomandibular disorder. Oral health of the patients was examined before treatment, and their acute problems were eliminated by basic dental treatments before participation in the study. 

Sleep study with a baseline overnight polysomnography (PSG) in sleep laboratory revealed an obstructive apnea/hypopnea index (AHI) between 5 and 40 events per hour during patients' sleep (including no central or mixed OSA patients). The first half of 24 patients, who refused and/or not tolerated the use of nCPAP device, had mild OSAS, whereas the second half of the patients had moderate OSAS (AHI of 5 to 20 is considered mild, and 21 to 40 is moderate according to the American Academy of Sleep Medicine) [9].

**Figure 1:**
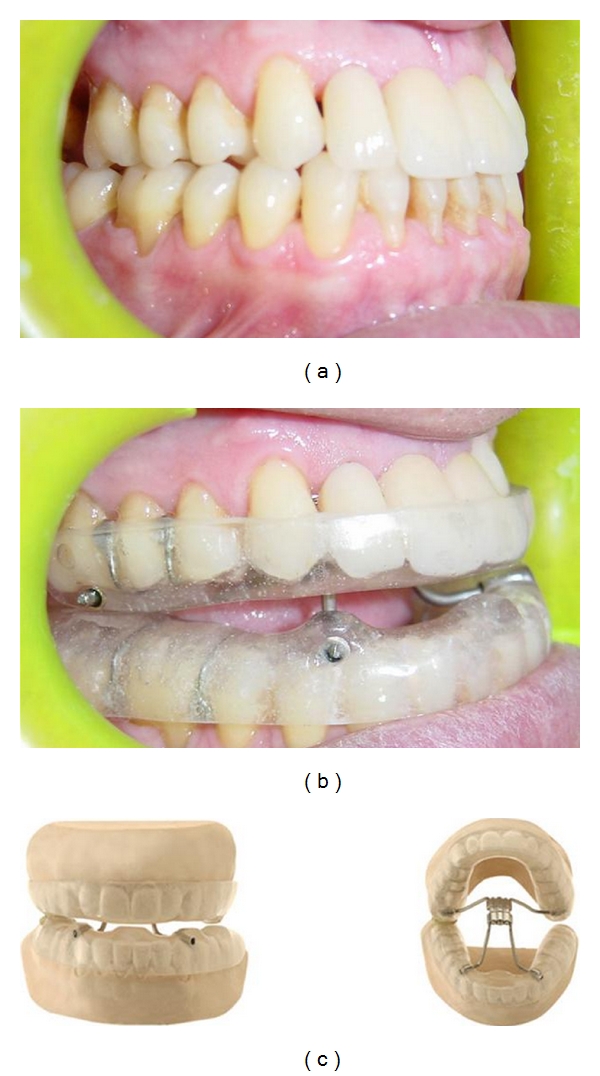
Titratable appliance (Klearway).

**Figure 2:**
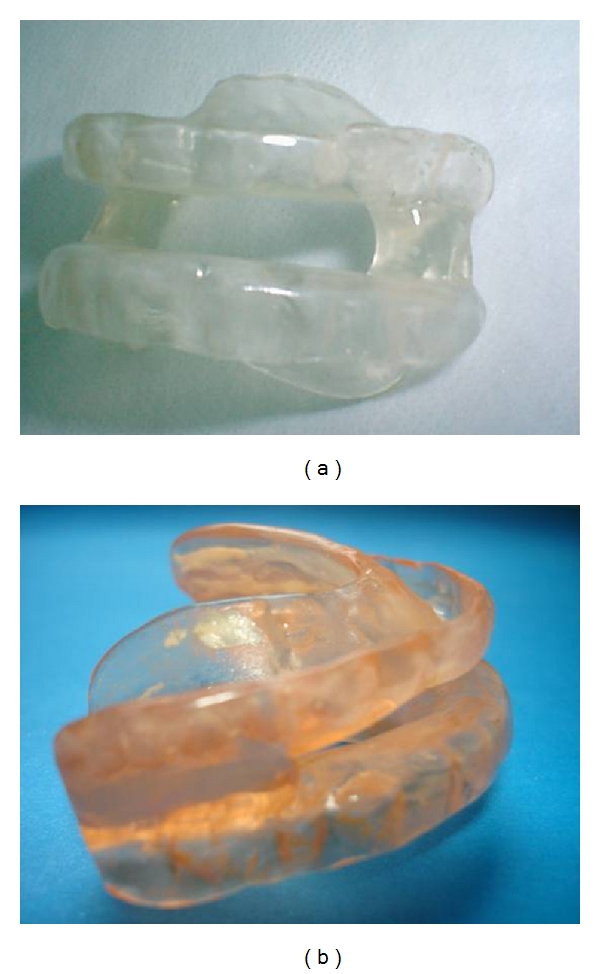
Mandibular advancement splint (MAS).

In the study, 24 OSA patients were divided into two groups (group KW and group MAS). In the group KW, twelve subjects, including 6 mild (KW-mi) and 6 moderate (KW-mo) OSAS, used Klearway appliance with 5mm vertical dimension (Figure 1) [10, 14]. Klearway appliance had an expansion screw in the palate. The dentist advanced the appliance by 0.5 mm increments per week. It was fabricated from thermoactive acrylic, increasing retention and decreasing tooth discomfort. It also permitted lateral and vertical jaw movements. The other 12 subjects, including 6 mild (MAS-mi) and 6 moderate (MAS-mo) OSASs, received MAS with 75% protrusion of the mandible and 5 mm vertical dimension (group MAS) (Figure 2). Mandibular advancement splint was made from rigid acrylic. The ventilation hole was placed in the anterior portion of the appliances to allow easy respiration throughout the night [2, 6, 8, 11, 15]. 

Informed consent was obtained from all patients. The experiments were conducted in accordance with the principles of the Declaration of Helsinki for Human Experimentation.

### 2.2. Protocol (data collection)

Five nighttime polysomnograms were performed.

(0.PSG): All patients underwent first-night polysomnogram without using any appliance.
(PSG. K1): The first week of control Polysomnogram performed to Group K
(PSG. K2): Third control polysomnogram after one month of Klearway usage.
(PSG. M1): The first week of control polysomnogram performed to group M.
(PSG. M2): Third control polysomnogram after one month of MAS usage.

All subjects completed the Epworth Sleepiness Score (ESS) before the usage of appliances and at the end of the month during which the appliances were used.

### 2.3. Statistical Analysis

The data were analyzed by using SPSS software (SPSS, Chicago, III). Within-group differences before and after the appliance usage were evaluated by the Wilcoxon signed-ranks test. 

As a result of the 3-way ANOVA analysis made to compare the results of AHI among the different time intervals in all groups, statistical differences at a level of *P* < .05 were found. In order to determine from which time interval this difference came from, Tukey HSD test was used. Statistical differences were evaluated in terms of *P* < .05.

## 3. Results

Each subject underwent 3 sleep studies after the usage of each appliance. Adjustment of the K appliance was continued until the subjective signs and symptoms of OSAS were reduced or disappeared. Three of the 12 subjects required no advancement beyond 75% maximum protrusion position in group KW. The mean total amount of mandibular advancement was 9.4 ± 1.3 mm (85%), and vertical dimension between the mandible and maxilla was 5 mm.

The MAS appliance was fabricated with a position of 75% of maximum protrusion and 5mm vertical dimension. Three of the 12 patients in group MAS and one of the 12 patients in group KW described mild pain in the TMJ and muscle tenderness. However, it improved to be only slightly uncomfortable over time. On the other hand, gum irritation was only observed in three patients of group MAS. Two patients of group KW complained about lower anterior tooth discomfort in the morning. However, these complaints in both group KW and MAS disappeared after a week.

The median initial ESS reduced from 11.25 ± 0.7 to 4.7 ± 1.0 in group KW at the end of the first month (*P* < .001). Reduction in ESS was also observed at the end of the first month from 11.1 ± 1.7 to 4.5 ± 0.9 in group MAS (*P* = .003 < .01). All subjects subjectively reported more restful sleep with reduction of snoring both at the end of the first week and the first month.

Polysomnographic data results as Mean±SD and statistical significance are shown in Tables 1 and 2. The mean AHI statistically decreased in both groups (*P* < .05). Using PSG data, there was no change in total sleep time, but the mean REM time during sleep increased in both the groups the both at the end of the first week and the first month (*P* < .05). However, there was no change in non-REM time during sleep, or time spent in the supine position while asleep in both the groups. In addition, the mean oxygen saturation (SaO_2_) improved in the both group KW and Group MAS at the different time intervals (*P* < .05).

Comparison of the AHI at different time intervals for each group is shown in Table 3. Statistically significant differences were found between the baseline and first week, baseline and first month in mean values of AHI for KW-mi, KW-mo, MAS-mi, and MAS-mo groups (*P* < , 05). Besides, the means of AHI for KW-mo and MAS-mo groups at the end of the first week and the first month were found to be statistically different (*P* < , 05) in favour of the KW group (Table 3).

 The comparison between KW (KW-mi, KW-mo)and MAS (MAS-mi, MAS-mo) groups of the mean AHI results at the end of first month and first week has been given in Table 4. Accordingly, the decrease in AHI of the KW-mo group at the end of the 1st month was determined to significantly greater different than that of the MAS-mo group (*P* = .019 < .05).

**Figure 3:**
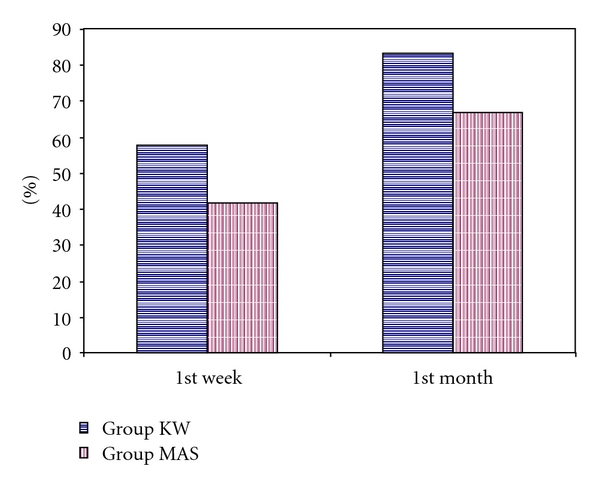
The success rates (%) of the groups at the end of the first week and first month.

The treatment success was defined by resolution of symptoms such as reduction in AHI <15 events/hr or at least 50% reduction from baseline AHI [9]. The success rate at the end of the first week and first month was shown in Figure 3 for both groups. Reduction of AHI to <15 events/hr was observed in 7 of the 12 patients (58%) at the end of the 1st week and 10 of the 12 patients (83.3%) at the end of the 1st month in group KW. The treatment success in 5 of 12 patients (42%) at the end of the 1st week and 8 of the 12 OSA patients (66.7%) at the end of the 1st month was observed in MAS treatment group in our study.

## 4. Discussion

In this study, the effect of Klearway and MAS appliances on OSA patients at end of the first week and first month after the appliance insertion was evaluated. The results indicated that both appliances were effective in the treatment of snoring and OSAS. Following Hans et al.'s [17] suggestion that future randomized clinical trials of dental devices for treatment of OSAS and snoring should not use a placebo device as one of the treatment groups, we did not use a control group or a placebo device, 

The differences between the two appliances were method of retention and construction, flexibility of material, adjustability, freedom of jaw movement, and quantity of the mandibular protrusion. Both appliances were well tolerated by all subjects. Although several side effects including excessive salivation, dryness of the mouth, and bruxism were observed in both groups, these side effects improved in a week. Gum irritation was observed in 3 patients of group MAS, but not in group KW. It could be the result of the thermoelastic material the Klearway appliance was made of. Tooth discomfort was seen in two patients of group KW. This could be due to the increased retention of the Klearway appliance. Some clinicians find increased effectiveness when the lower jaw is rigidly stabilized like in the MAS [15], and others feel that a slight degree of mobility enhances TMJ comfort like in Klearway [10, 14]. Three of the 12 patients in group MAS and 1 of the 12 patients in group KW described mild pain around TMJ and muscle tenderness, respectively. This complaints disappeared over time. De Almeida et al. [18] had reported that OAs were safe regarding to TMJ alterations in the treatment of patients with OSAS for one year. 

All patients subjectively reported more restful sleep with a reduction of snoring. Indeed, initial EES score dropped at the end of the first month in both groups. On the other hand, PSG data showed that mean REM time increased, and there was no change in non-REM time during sleep in both groups both at the end of the first week and the first month while there was no change in total sleep time. In addition, minimum oxygen saturation increased at the end of the first week and also increased above 90% oxygen saturation at the end of the first month in both groups. 

 Optimal treatment of OSAS would result in abolition of apnea and hypopnea, as can be achieved with nCPAP. However, treatment might be considered adequate with reduction of AHI below some defined level. Mortality with OSAS has been found to be significantly increasing where the number of apneas per hour of sleep exceeds twenty. In the current study, the treatment success was defined by a resolution of symptoms, either by reduction of AHI to less than 15 events/hr or by at least a 50% reduction of AHI compared to the baseline values [9, 18, 19]. Different studies using Klearway reported a treatment efficiency ranging from 50% to 80% [10, 14, 18, 20, 21]. Reduction of AHI to <15 events/hr was observed in 58% of patients at the end of the 1st week and 83.3% of patients at the end of the 1st month in group KW. These results coincide with those of Lowe et al. [14] who reported that 80% success rates were achieved in mild OSA patients and 60% in more severe OSA patients. On the other hand, reduction of AHI to <15 events/hr and relief of symptoms were evaluated as a treatment success in 42% of patients at the end of the 1st week and 66.7% of patients at the end of the 1st month in MAS treatment group in our study. However, no statistically significant difference of the treatment success was found between the KW and MAS group at the end of the first week and the first month (*P* > .05). These findings suggested that K and MAS appliances had potentially useful role in the treatment of OSA patients similar to each other. Our results were similar with the previous studies [13, 14, 20]. On the other hand, the reason for high success rates of both group KW and group MAS patients at the end of the 1st month compared to those of the 1st week could be explained by getting accustomed to the usage of appliances. Additionally, a decrease was observed in the mean AHI results of the KG-mo and MAS-mo at the end of the first month when compared with AHI results of the first week. 

 No statistical difference was found between the number of patients in KW-mi and MAS-mi who reached AHI reduction <15 events/hr, both at the 1st week and the 1st month. Although no statistically significant changes in AHI were observed between the KW-mo and MAS-mo in the 1st week (*P* > .05), AHI was found to be statistically different for KG-mo and MG-mo at the 1st month (*P* < .05). That is why MAS should be offered as the treatment of first choice in relatively mild OSA patients, while the Klearway appliance can be recommended both to mild and moderate OSA patients. However, in previous studies, mandibular advancement appliances were proposed to mild and moderate OSA patients [13, 19]. According to our findings, we may recommend MAS appliance only to mild OSA patients. 

The correct therapeutic position of the mandible is critical for the success of appliance in OSA patients, both in terms of opening the airway as well as patient comfort and compliance [5, 19, 22, 23]. Meurice et al. [23] reported that mouth opening was effective in pharyngeal collapsibility. However, Pitsis et al. [24] recently showed that altering the amount of bite opening by a mandibular advancement device did not alter its polysomnographic effects in patients with OSA. Both MAS and Klearway were designed with 5 mm vertical opening and were found to be successful on the OSA patients of this study. 

De Almeida et al. [18] evaluated the relationship between different increments of mandibular protrusion and AHI after the insertion of titratable OA (Klearway). They found that the reduction in the AHI depended on the amount of mandibular protrusion. Lamont et al. [19] designed two different types of MAS. Type A device produced a maximum mandibular protrusion with 3-4 mm interincisal opening, while type B permitted up to 70% mandibular protrusion with 6–9 mm vertical opening. They found that type B device was more effective on AHI than type A. The more reduction of mean AHI in the KG-mo in comparison to the reduction of those of MG-mo at the end of the first month could be attributed to the potential of Klearway appliance to yield the desired amount of mandibular advancement. However, the advancement of mandible was 75% of the maximum mandibular protrusive capacity in group MAS. Kuna et al. [16] suggested that if mandible was advanced to 85.2 ± 25.8% of maximum voluntary protrusion by Klearway appliance during treatment in OSA patients, acceptable reduction in AHI could be obtained. Klearway appliance had more advantage versus MAS appliance since the mandible advanced slowly over time and allowed bone and soft tissue structure to adjust this displacement in the meantime. On the other hand, during the two years of OA treatment in OSA patients, anatomic and occlusal changes were shown by Bondemark [25]. Thus, two-year followup study to observe the changes in TMJ anatomy during the AHI reduction therapies must be done in the future. 

There were method limitations in this study. The sample size was small, and extrapolation of the results to a larger population might be questionable for several reasons. The effects of long-term alteration in mandibular posture, TMJ, and the oral tissues need to be investigated. The morbidity associated with OSAS is of a chronic nature. Successful management of chronic disease requires therapy that is effective over a period of years rather than a week or month.

## 5. Conclusion

Klearway and MAS appliances are both effective in the treatment of mild and moderate OSA patients. 

An appliance (Klearway) that provides advancement of 85% of mandibular protrusion to open the upper airway was more effective in reducing the number of high apneic events during sleep than one (MAS) which provides 75%. 

Mandibular advancement splint (MAS) should be preferred in mild OSA patients rather than moderate OSA patients.

## Figures and Tables

**Table 1:** Polysomnographic results of Klearway appliance group.

NORMS (*N* = 12)	0.PSG	PSG.K1	0.PSG-PSG.K1	PSG.K2	0.PSG-PSG.K2	PSG.K1-PSG.K2
			*P*		*P*	*P*
Total sleep time (mins.)	227.6 ± 101.9	289.2 ± 87.5	NS	253.5 ± 75.5	NS	NS
AHI (total sleep)	18,8 ± 7,3	10.0 ± 4.3	.000***	7.3 ± 3.3	.000***	.001***
sleep efficiency	82.0 ± 9.3	88.2 ± 8.9	.001***	86.0 ± 7.2	<001***	.001***
Minimum SaO_2_	72.0 ± 7.9	84.7 ± 7.6	<001***	91.2 ± 6.1	<001***	<001***
NREM (mins)	27.2 ± 9.2	22.3 ± 7.2	NS	25.5 ± 8.2	NS	NS
REM (mins)	63.4 ± 18.2	82.5 ± 24.5	<001***	88.2 ± 20.8	<001***	<001***

(0.PSG): First-night polysomnogram without using any appliance so as the baseline severity of OSA to be determined.

(PSG.K1): The first week of control polysomnogram performed to group KW following 1-week insertion of the Klearway appliance.

(PSG.K2): Third control polysomnogram after one-month usage of Klearway appliance.

*N* = 12 Mean ± SD, mins = minutes, AHI = Apnea-hypopnea index, SaO_2_ = Minimum oxygen saturation, REM = Rapid eye movement, NREM = Nonrapid eye movement;

*P < .05. **P < .01. ***P < .001.

**Table 2:** Polysomnographic results of mandibular advancement splint (MAS) group.

NORMS (*N* = 12)	0.PSG	PSG.M1	0.PSG-PSG.M1	PSG.M2	0.PSG-PSG.M2	PSG.M1-PSG.M2
			*P*		*P*	*P*
Total sleep time (mins.)	226.3 ± 106.2	272 ± 92.4	NS	253.5 ± 88.5	NS	NS
AHI (total sleep)	17.9 ± 6.8	10.0 ± 4.5	.000***	9.1 ± 4.9	.009***	.009***
Sleep efficiency	79.0 ± 8.9	87.4 ± 8.0	.001**	88.0 ± 9.2	<001***	<001***
Minimum SaO_2_	71.0 ± 6.3	88.2 ± 6.5	<001***	90.1 ± 7.8	<001***	.01*
NREM (mins)	25.2 ± 8.1	26.0 ± 8.9	NS	22.2 ± 7.3	NS	NS
REM (mins)	61.2 ± 16.5	83.2 ± 19.8	.01*	87.4 ± 22.8	<001***	.001**

(0.PSG): First-night polysomnogram without using any appliance so as the baseline severity of OSA to be determined.

(PSG.M1): The first week of control polysomnogram performed to group MAS following 1-week insertion of the mandibular advancement splint appliance.

(PSG.M2): Third control polysomnogram after one-month usage of MAS appliance.

*N* = 12 Mean±SD, mins= minutes, AHI = Apnea-Hypopnea Index, SaO_2_ = Minimum oxygen saturation, REM = Rapid eye movement, NREM = Nonrapid eye movement;

*P < .05. **P < .01. ***P < .001.

**Table 3:** The comparison of the AHI at different time intervals for each groups. SE: Standard Error. Significance level *α* =.05.

Groups	0.PSG—1-week*-*PSG mean difference ± SE (AHI)	*P*	0.PSG—1-month-PSG mean difference ± SE (AHI)	*P*	1-week*-*PSG—1-month-PSG mean difference ± SE (AHI)	*P*
KW-mi	5.55 ± 0.8250	***.000***	6.75 ± 0.8250	***.000***	1.25 ± 0.8250	.266
KW-mo	12.083 ± 0.9742	***.000***	16.333 ± 0.9742	***.000***	4.2500 ± 0.9742	***.001***
MAS-mi	4.41675 ± 0.7662	***.000***	6.333 ± .07662	***.000***	1.9167 ± 0.7662	,089
MAS-mo	9.667 ± 0.9036	***.000***	11.1667 ± 0.9036	***.000***	1.500 ± 0.9036	***,046***

**Table 4:** Comparison of the AHI between groups at different time intervals. Significance level *α* =.05.

Time	KW-mi (AHI)	MAS-mi (AHI)	KW-mi/MAS-mi (AHI)	KW-mo (AHI)	MAS-mo (AHI)	KW-mo/MAS-mo (AHI)
			*P*			*P*
1st week	6.6 ± 2.2	7.2 ± 2.1	.552	13.5 ± 3.0	14.5 ± 3.0	.43
1st/month	5.4 ± 3.0	5.3 ± 2.7	.944	9.2 ± 2.13	13.0 ± 3.3	***.019***
